# EMBL’s European Bioinformatics Institute (EMBL-EBI) in 2022

**DOI:** 10.1093/nar/gkac1098

**Published:** 2022-12-07

**Authors:** Matthew Thakur, Alex Bateman, Cath Brooksbank, Mallory Freeberg, Melissa Harrison, Matthew Hartley, Thomas Keane, Gerard Kleywegt, Andrew Leach, Mariia Levchenko, Sarah Morgan, Ellen M McDonagh, Sandra Orchard, Irene Papatheodorou, Sameer Velankar, Juan Antonio Vizcaino, Rick Witham, Barbara Zdrazil, Johanna McEntyre

**Affiliations:** Data Services Teams, EMBL’s European Bioinformatics Institute (EMBL-EBI), Wellcome Genome Campus, Hinxton CB10 1SD, UK; Data Services Teams, EMBL’s European Bioinformatics Institute (EMBL-EBI), Wellcome Genome Campus, Hinxton CB10 1SD, UK; Data Services Teams, EMBL’s European Bioinformatics Institute (EMBL-EBI), Wellcome Genome Campus, Hinxton CB10 1SD, UK; Data Services Teams, EMBL’s European Bioinformatics Institute (EMBL-EBI), Wellcome Genome Campus, Hinxton CB10 1SD, UK; Data Services Teams, EMBL’s European Bioinformatics Institute (EMBL-EBI), Wellcome Genome Campus, Hinxton CB10 1SD, UK; Data Services Teams, EMBL’s European Bioinformatics Institute (EMBL-EBI), Wellcome Genome Campus, Hinxton CB10 1SD, UK; Data Services Teams, EMBL’s European Bioinformatics Institute (EMBL-EBI), Wellcome Genome Campus, Hinxton CB10 1SD, UK; Data Services Teams, EMBL’s European Bioinformatics Institute (EMBL-EBI), Wellcome Genome Campus, Hinxton CB10 1SD, UK; Data Services Teams, EMBL’s European Bioinformatics Institute (EMBL-EBI), Wellcome Genome Campus, Hinxton CB10 1SD, UK; Data Services Teams, EMBL’s European Bioinformatics Institute (EMBL-EBI), Wellcome Genome Campus, Hinxton CB10 1SD, UK; Data Services Teams, EMBL’s European Bioinformatics Institute (EMBL-EBI), Wellcome Genome Campus, Hinxton CB10 1SD, UK; Data Services Teams, EMBL’s European Bioinformatics Institute (EMBL-EBI), Wellcome Genome Campus, Hinxton CB10 1SD, UK; OpenTargets, EMBL’s European Bioinformatics Institute (EMBL-EBI), Wellcome Genome Campus, Hinxton CB10 1SD, UK; Data Services Teams, EMBL’s European Bioinformatics Institute (EMBL-EBI), Wellcome Genome Campus, Hinxton CB10 1SD, UK; Data Services Teams, EMBL’s European Bioinformatics Institute (EMBL-EBI), Wellcome Genome Campus, Hinxton CB10 1SD, UK; Data Services Teams, EMBL’s European Bioinformatics Institute (EMBL-EBI), Wellcome Genome Campus, Hinxton CB10 1SD, UK; Data Services Teams, EMBL’s European Bioinformatics Institute (EMBL-EBI), Wellcome Genome Campus, Hinxton CB10 1SD, UK; Data Services Teams, EMBL’s European Bioinformatics Institute (EMBL-EBI), Wellcome Genome Campus, Hinxton CB10 1SD, UK; Data Services Teams, EMBL’s European Bioinformatics Institute (EMBL-EBI), Wellcome Genome Campus, Hinxton CB10 1SD, UK; Data Services Teams, EMBL’s European Bioinformatics Institute (EMBL-EBI), Wellcome Genome Campus, Hinxton CB10 1SD, UK

## Abstract

The European Molecular Biology Laboratory's European Bioinformatics Institute (EMBL-EBI) is one of the world's leading sources of public biomolecular data. Based at the Wellcome Genome Campus in Hinxton, UK, EMBL-EBI is one of six sites of the European Molecular Biology Laboratory (EMBL), Europe's only intergovernmental life sciences organisation. This overview summarises the status of services that EMBL-EBI data resources provide to scientific communities globally. The scale, openness, rich metadata and extensive curation of EMBL-EBI added-value databases makes them particularly well-suited as training sets for deep learning, machine learning and artificial intelligence applications, a selection of which are described here. The data resources at EMBL-EBI can catalyse such developments because they offer sustainable, high-quality data, collected in some cases over decades and made openly availability to any researcher, globally. Our aim is for EMBL-EBI data resources to keep providing the foundations for tools and research insights that transform fields across the life sciences.

## INTRODUCTION

The European Molecular Biology Laboratory's European Bioinformatics Institute (EMBL-EBI) is one of the world's leading sources of public biomolecular data. Based at the Wellcome Genome Campus in Hinxton, UK, EMBL-EBI is one of six sites of the European Molecular Biology Laboratory (EMBL), Europe's only intergovernmental life sciences organisation, whose world-class research infrastructure and services support cutting-edge science globally.

EMBL-EBI enables life science research and its translation to medicine, agriculture, industry and society by:

- freely providing data and bioinformatics services to the scientific community in ways that promote scientific progress.
- contributing to the advancement of biology through investigator-driven research.
- providing bioinformatics training to scientists at all levels.
- disseminating cutting-edge technologies to industry and applications of science.
- supporting, as an ELIXIR Node, the coordination of biomolecular data provision in Europe.

EMBL-EBI contributes to EMBL’s 2022–2026 ‘Molecules to Ecosystems’ programme, which aims to establish the molecular basis of life in context, to gain new knowledge that is relevant to understanding life on Earth, and to provide translational potential to support advances in human and planetary health

This overview focuses on services that EMBL-EBI data resources provide to scientific communities globally, describing related training and industry applications where relevant. As many other EMBL-EBI data resources have dedicated articles elsewhere in this special issue, this overview focuses primarily on major changes to data resources not described elsewhere.

EMBL-EBI data resources comprise: deposition databases, which archive experimental data; added-value databases, which provide annotation, curation, reanalysis and integration of deposited data; and open source software tools, that enable reuse of these resources. Deposition databases, added-value databases and tools are described and accessed via the EMBL-EBI services web portal. All EMBL-EBI data resources and many software systems can be downloaded and installed locally, and are made available on an open and free basis for reuse. Many services offer further bulk and machine-readable access including via API, FTP, Aspera and Globus services.

Co-housing these data resources at one institute results in close integration between resources, demonstrated by the high degree of between-resource data flow, and the availability of integrated tools like pan-resource EBI-Search (1). Data resources also benefit from institutional support with technical and infrastructure management. EMBL-EBI resources serve as foundations for hundreds of external resources and tools (with many recent developments described below). Europe's flagship bioscience data coordination programme ELIXIR identifies the Core Data Resources and Deposition Databases of most fundamental importance to the wider life-science community and the long-term preservation of biological data. Many EMBL-EBI resources have achieved this designation.

The scale, openness, rich metadata and extensive curation of EMBL-EBI added-value databases makes them particularly well-suited as training sets for deep learning, machine learning and artificial intelligence applications (abbreviated here under the umbrella term AI applications). A recent major AI application is the DeepMind AlphaFold system for predicting previously unknown 3D structures, which was trained on openly available experimentally verified protein structure data from the Protein Databank (2), jointly delivered by EMBL-EBI and other partners (the wwPDB consortium), as well as protein sequences and annotation from Uniprot (3) and metagenomics data from MGnify (4). As of September 2022, the outputs of AlphaFold, hosted by EMBL-EBI as AlphaFold-DB (5), included 214 684 311 predicted structures, with 48 complete proteomes available for bulk download. Over 500 000 researchers in 190 countries used AlphaFoldDB in its first year of operation. The data is already enabling researchers to progress a number of previously intractable research questions (6). Further examples of how EMBL-EBI resources are enabling AI applications in proteomics, drug discovery, imaging and other areas are included below.

### The impact of EMBL-EBI data resources

EMBL-EBI tracks the use of data resources through metrics including the number of web requests and unique IP addresses visiting service websites, the volume of data deposited, and the number of open citations EMBL-EBI data resources receive in scientific publications. While each metric has limitations and cannot provide an exact quantification of use, considered together they give an indication of the scale and trend in usage.

**Figure 1.**
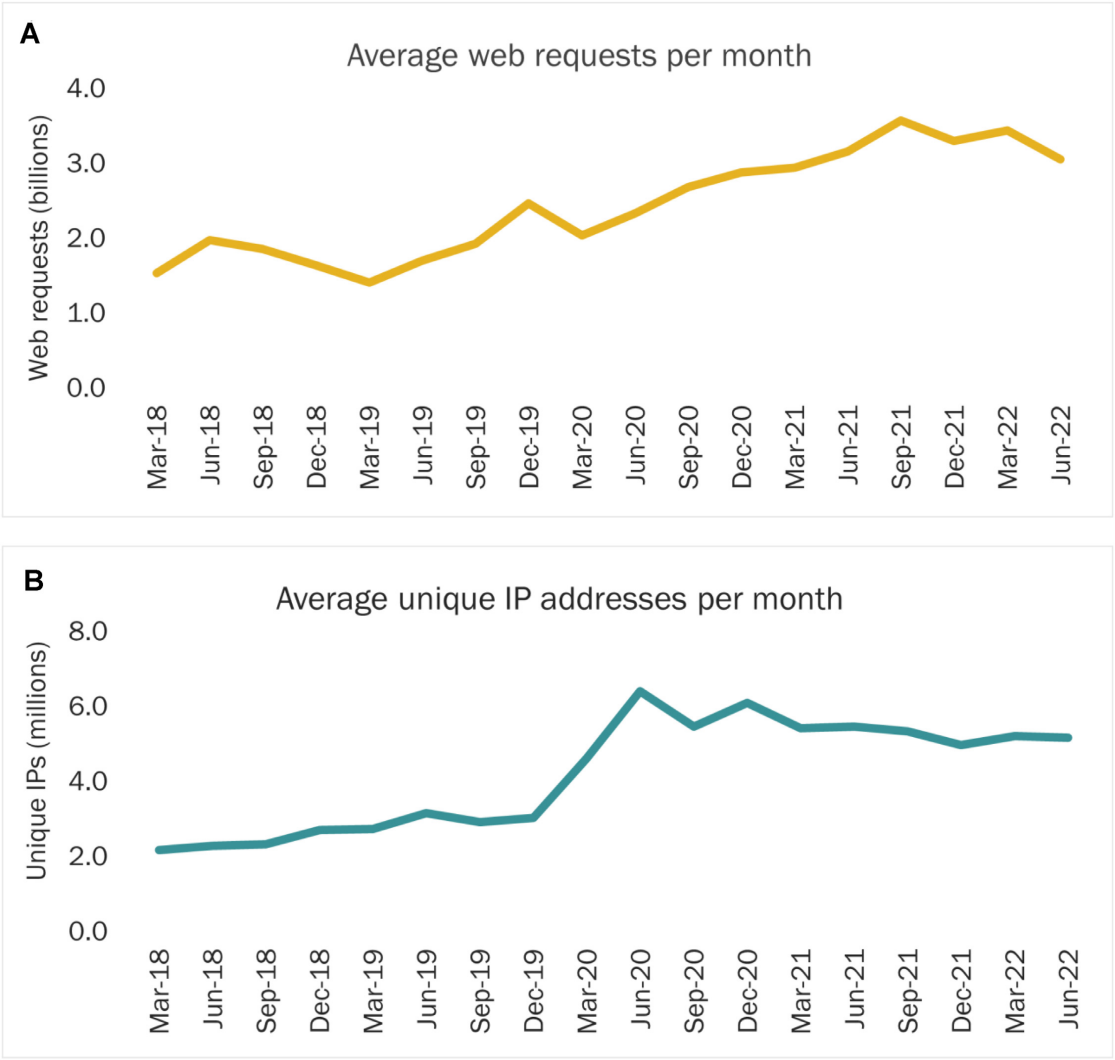
Web requests (yellow, **A**) and Unique IP visits (blue, **B**) to EMBL-EBI data resources, 2018–2022.

Demand from researchers for EMBL-EBI data resources increased considerably in 2020, particularly during the second quarter (April–June 2020) which coincided with the beginning of the global COVID-19 pandemic. Usage continued to grow throughout the rest of 2020 and into 2021, as many researchers transitioned back from remote to in-person or hybrid working (Figure 1). Demand has remained high in the first two quarters of 2022 with an average of 3.1 billion web requests and 5.2 million unique IPs per month in the second quarter of the year. This is ∼100% higher than user demand for the equivalent period in 2018. EMBL-EBI data resources have a global reach, with every UN member state country represented in our user base in 2021, and the data available for the current year to date suggests similar global user demand in 2022.

**Figure 2.**
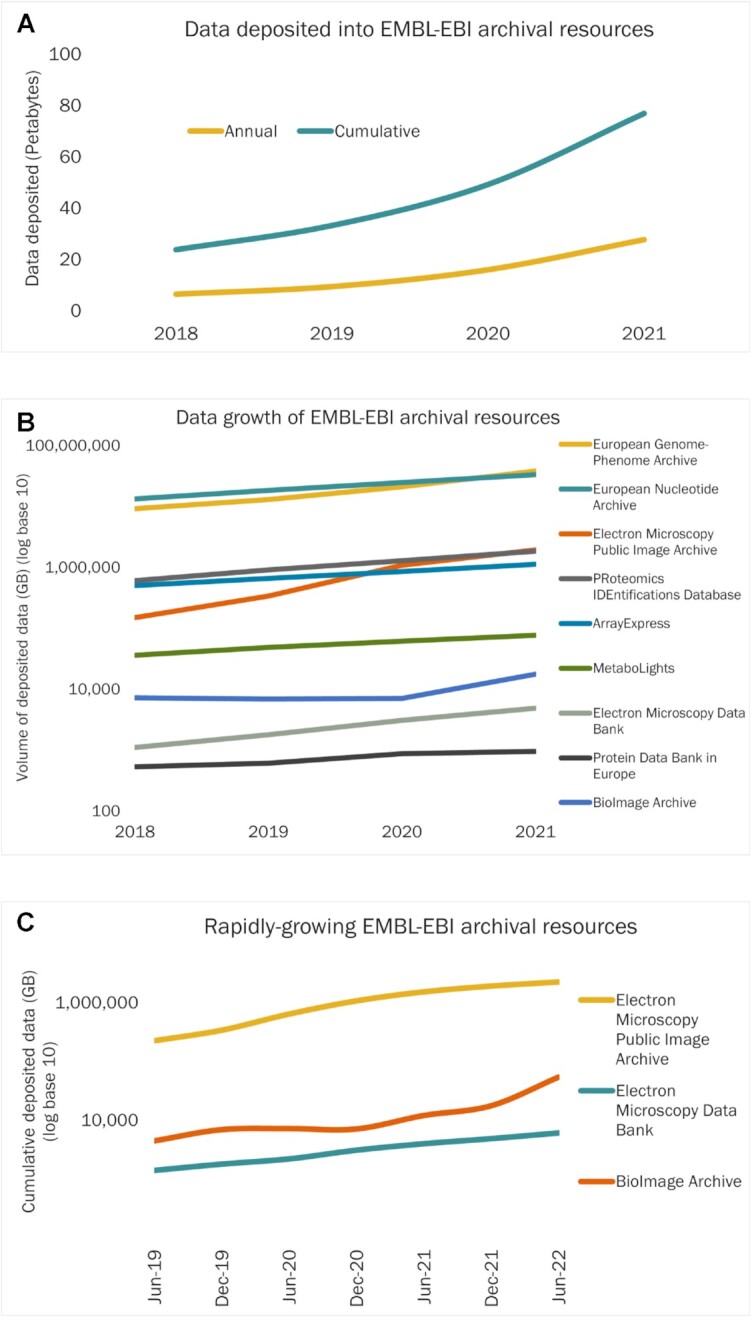
(**A**) Annual (yellow) and cumulative (blue) data deposition into EMBL-EBI archival data resources. (**B**) Annual deposition into nine archival resources. Note the logarithmic scale. and rapid rate of growth for the Imaging and cryo-electron microscopy resources Bioimage Archive, EMPIAR and EMDB. (**C**) Annual quarterly data growth for the Bioimage Archive, EMPIAR and EMDB imaging and cryo-electron microscopy data resources. Note the logarithmic scale.

The rate of data deposition by volume into EMBL-EBI’s archival resources continues to accelerate, with over 25 PB of data deposited in 2021, bringing the cumulative total storage up to approximately 75 Petabytes (Figure 2). The two largest archival resources are European Nucleotide Archive (ENA) (7) and European Genome-phenome Archive (EGA) (8), between them accounting for over 90% of total data deposited to date. Notably rapid data growth in recent years has been in imaging data resources - BioImage Archive (BIA) (9); and the electron microscopy imaging resources Electron Microscopy Public Image Archive (EMPIAR) (10) and Electron Microscopy Databank (EMDB) (11)

In 2021, an independent study estimated the economic value and impact of EMBL-EBI data resources. The study found that researchers spent 140 million hours/year using EMBL-EBI data resources, with a value equivalent to £5.5 billion.

## MAJOR CHANGES IN THE EMBL-EBI DATA RESOURCE PORTFOLIO

### Federated EGA network officially launched

Until recently, most of the individual-level human omics data made discoverable on the EMBL-EBI European Genome-phenome Archive (EGA) (8) were generated by research consortia, not in healthcare settings. Many countries now have personalised medicine programmes that are generating data from national or regional initiatives, resulting in a shift from research-driven to healthcare-driven genomic data. Data generated in a healthcare context can be subject to different governance and national data protection legislation than research data, and these access controls risk blocking reuse for research. If reuse for research were not possible, the potential value and impact of emerging healthcare genomic data would be significantly reduced. Federated EGA uses a distributed network of international repositories to ensure genomic data accelerates research by enabling transnational discovery of and access to human data, while also respecting jurisdictional data protection regulations, thus enabling scale and more powerful research insights.

One of the first applications of Federated EGA is to provide transnational data discovery and access infrastructure for the European 1+ Million Genomes and Genome Data Infrastructure projects. The subsequent Beyond One Million Genomes EC coordination and support actions project will demonstrate how Federated EGA enables rare disease federated discovery and access.

The Federated EGA network was officially launched in 2022 with the signing of the first legal agreements with inaugural nodes in Sweden, Norway, Germany, Finland, and Spain. Dozens of additional nodes across Europe and the world are working towards joining the Federated EGA network, with a shared vision of establishing a truly global resource for sensitive human data discovery and sharing.

### Pfam merging into InterPro

EMBL-EBI hosts two major protein family resources, Pfam (12) and InterPro (13). The Pfam database of protein families was formerly hosted at the Sanger Institute until 2012, when it was migrated to EMBL-EBI. Although similar in scope, there are important differences between the two resources. InterPro provides a comprehensive view across most of the world's protein family resources by aggregating data from 13 other resources, including Pfam (13). Although it brings protein family data together it does not generate the signatures for identifying a particular protein family, such as a profile-hidden Markov model. The signatures are provided by the 13 member databases. Pfam provides EMBL-EBI the ability to create new family signatures as well as update existing ones and thus provides an important complementary functionality to InterPro. To make the production and dissemination of these two resources as efficient and scalable as possible, the functionality of the Pfam website was merged into Interpro. The Pfam website was decommissioned in January 2023, but all of its data and functionality continue to be provided via InterPro.

### ArrayExpress migrating into BioStudies

The BioStudies Database (14) is a resource for encapsulating all the data associated with a biological study, which may exist across a number of different data resources. One of the goals of BioStudies is to manage data generated in experiments that can be characterized as ‘multi-omics’. Increasingly, many experiments that used to belong to the domains of transcriptomics or functional genomics are now multi-modal, resulting in decreased depositions into the array-specific ArrayExpress data resource (15). Since 2020, to streamline the data submission processes and data representation at EMBL-EBI, data served from ArrayExpress has been migrated to BioStudies, under the ‘ArrayExpress collection’. Following positive user feedback on the new pipelines and processes, the ArrayExpress interface was decommissioned in September 2022 and all new functional genomics submissions are now processed and loaded in BioStudies, before flowing on into other data resources including ENA [7].

## NEW FEATURES AND AI APPLICATIONS OF EXISTING DATA RESOURCES

### UniProt annotates 50 million previously uncharacterised proteins using machine-learning

The UniProt Knowledgebase of protein sequence and function (16) combines both automated and expert-curated annotations of protein function. Expert biocurators link UniProtKB/Swiss-Prot entries to a summary of experimentally verified, or computationally predicted, functional information about each protein. Information is added to non-reviewed entries in the UniProtKB/TrEMBL system using annotation transfer from reviewed entries by automated systems (17).

A UniProt-organised challenge in 2022 asked competitors from the machine learning community to develop software tools and algorithms which predict metal binding sites in proteins with accuracy and at scale. The best of these tools will be incorporated into future production pipelines.

UniProt data is made available in formats suitable for researchers to develop their own tools and resources. As of release 22_05, sequence embeddings for all of UniProtKB/Swiss-Prot are released on the UniProt ftp site. Longer-term plans are to make records more readily machine-readable, for example by increasing usage of ontologies, to support utility as positive training sets in AI applications.

### Mass spectrometry-based proteomics datasets drive AI applications

Proteomics Identification Database (PRIDE) is the world-leading database for mass spectrometry (MS)-based proteomics datasets (18) and is one of the founding members of the International ProteomeXchange Consortium of proteomics resources (19). On average, ∼500 datasets were submitted to PRIDE every month in 2022. The unprecedented availability of proteomics datasets in the public domain is driving multiple applications reusing this data. AI applications have been applied to improve every step in the proteomics analytical workflow—for a recent review see (20). These approaches are enabling new biological findings, for instance in the context of protein phosphorylation (21), identification of antimicrobial peptides (22) and prediction of antigen presentation of HLA molecules (23). Multi-omics approaches involving proteomics data are an area where further applications of public datasets will generate novel tools. All PRIDE datasets that have been used for AI applications, including training and evaluating models, are tagged using the term ‘Machine Learning’. This shows the enormous value of public datasets with high quality annotation to enable novel ‘big data’ approaches in proteomics (24).

### Applications of small molecule bioactivity data for in silico drug discovery

The ChEMBL database (25) is a large-scale open resource of small molecule bioactivity data which was first launched in 2009. It mainly hosts curated data extracted from the medicinal chemistry literature as well as deposited datasets, and has grown significantly in size and complexity since its first release. The current release of ChEMBL (version 31, prepared in July 2022) hosts around 20 million bioactivity data points for 2.3 million compounds corresponding to 1.5 million assays, 15 000 targets, and 85 000 documents.

Prior to the launch of ChEMBL, only large private organisations were able to access diverse and high-quality (proprietary or commercial) bioactivity data sets for a wide range of biological targets at scale. Data from ChEMBL has proven indispensable for the development (26), validation and benchmarking (27–29) of a wide range of AI and other *in silico* applications, including those described below.

Given its vast size and coverage of medicinal chemistry space, ChEMBL is frequently leveraged for chemical space analysis, either driven by a focus on drugs (30), a chemotype-centric view (31,32), or a specific area of drug discovery research (33–35). ChEMBL also facilitates large-scale comparison of species differences in bioactivity data (36), and assay and bioactivity endpoint comparisons (37,38). Insights from such analyses influence how predictive models are built and applied, and guide experimental design when searching for new chemical matter.

ChEMBL facilitates the development of in silico target prediction algorithms (39–41) and molecular de novo design (42,43). Bioactivity data from ChEMBL, in conjunction with other data types such as pathway and disease information, is a foundational part of knowledge graph-based discovery tools, with applications such as phenotypic assay target deconvolution (44,45).

### AI-ready imaging datasets and interoperability standards

The BioImage Archive (9) is EMBL-EBI’s deposition database for life sciences imaging data associated with publications, as well as reference imaging datasets. AI applications are revolutionising the process of analysing and gaining insight from biological images. However, such techniques often generate ‘black box’ models. Understanding how these models function, what biases they may contain and what type of data they can be safely applied to, is very difficult without access to original training data.

To support reproducibility of AI applications in image analysis, the BioImage Archive supports deposition of both images and ground truth annotations used in training datasets. The archive already makes available over 30 imaging datasets with these AI-suitable annotations, which allows method developers to use existing data to accelerate development. Work is underway to enhance support for these ‘AI-ready’ datasets, through dedicated deposition pipelines, and developer friendly presentations. Bioimage Archive is playing an active role in the development of the community standards for interoperable segmentation, image categorisation and other annotation required to enable widespread imaging data sharing for AI applications (46).

The Electron Microscopy Public Image Archive EMPIAR (10), is a public resource for raw images underpinning 3D cryo-EM maps and tomograms (the latter archived in the Electron Microscopy Databank, EMDB (11)). EMPIAR also accommodates 3D datasets obtained with volume EM techniques, and soft and hard X-ray tomography. All data archived in EMPIAR can be re-used freely without any conditions or restrictions via a ‘CC0’ license model, making it an easily accessed source of data for AI applications in image analysis. EMPIAR released two datasets in 2022 specifically developed to support machine learning – CEM-MitoLab, a dataset of ∼22K cellular EM 2D images with label maps of ∼135K mitochondrial instances, and CEM1.5M: a cellular EM dataset containing ∼1.5M unlabeled 2D image patches curated for deep learning.

### Pre-print corpus for text-mining and AI applications

Europe Pubmed Central (Europe PMC) (47) provides open access to a worldwide collection of life science preprints and peer-reviewed journal articles. Following the COVID-19 full text preprints initiative, since April 2022 Europe PMC makes the full text of preprints supported by its 37 funders available for search, reading, and reuse, both on the Europe PMC website and programmatically in standard JATS XML format. As of September 2022 there were over 450 000 preprints indexed in Europe PMC from 24 preprint servers and nearly 32 000 of these are available as full text. Of the full text preprints 98% have an open access licence and are available via bulk download for text analytics and machine learning applications. To further possibilities for large-scale meta analyses, preprints in Europe PMC are linked to underlying research data, open peer review materials, citations, grants and other useful resources. The preprint corpus will increase discoverability, ensure the continued access to findings presented in preprints and enable new analytical possibilities including AI applications.

### Improving preprint transparency and tracking

The ability to improve and correct the manuscript through new versions is an important part of preprints’ appeal. However, changes to preprints can be difficult to track, especially across many different preprint servers and journals. Researchers working with preprints need to know which version should be cited, how a preprint differs from its published version, and whether conclusions presented in a preprint are valid after a preprint has been withdrawn or removed. To address these issues Europe PMC now offers a way to check for preprints updates. The **Article Status Monitor** is a Europe PMC tool that allows users to check if a preprint has been withdrawn, removed, published in a journal, or updated with a new version. Updates can be retrieved using a simple website tool, email alert or programmatically via the status-update-search module of the Articles API.

### Big data for target-disease association and disease-causing genes now available in the Cloud

The Open Targets consortium is a pre-competitive partnership between EMBL-EBI, the Wellcome Sanger Institute, and pharmaceutical company partners GSK, Sanofi, BMS—with Pfizer joining in 2022. The consortium generates data and builds informatics tools to enhance the identification and prioritisation of targets that will ultimately lead to more effective and safer drugs. Open Targets produce two open source informatics resources: the Open Targets Platform (48), which provides a knowledgebase and tools for target-disease association evidence and prioritisation; and Open Targets Genetics (49), developed to address the challenge of identifying disease-causing genes (and thus potential drug targets) from Genome-Wide Association Studies. These are increasingly being adopted as reference databases in their own right, but in addition provide structured data to enable other data integration and AI applications.

In May 2022, the resources were made available in the cloud via Google BigQuery and AWS Open Data. This integration and accessibility enables the data to be used in AI applications, such as machine learning to identify novel target-disease associations (50), building knowledge graphs for different biological insights (51–54) and for benchmarking new computational methods for drug target prioritisation (55,56).

**Figure 3.**
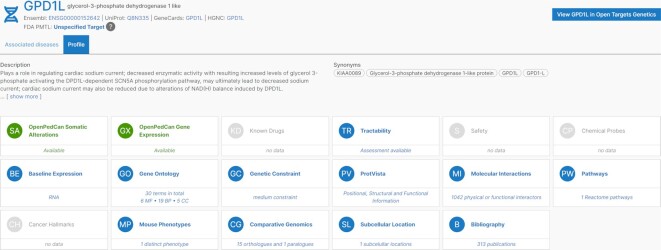
Example results from the Molecular Targets Platform, a US National Cancer Institute-supported instance of the Open Targets Platform with a focus on preclinical paediatric oncology data.

As the code base is open source, separate instances of the Platform can be created and adapted to user requirements—an example is the recent release of the NIH Childhood Cancer Data Initiative Molecular Targets Platform, which integrates tumor gene expression and somatic alteration data (Figure 3).

### Open data standards for proteomics

EMBL-EBI continues to lead many activities of the Proteomics Standards Initiative (PSI), the organisation in charge of developing open data standards in proteomics (57). Among these activities, during 2022, in collaboration with the Consortium for Top-Down Proteomics, the PSI released the ProForma 2.0 notation (58), providing a standard way to represent peptidoforms and proteoforms (combinations of protein sequences plus protein modifications).

ProForma can be used in conjunction with Universal Spectrum Identifiers (59), a PSI standard released in 2021 that provides a unique identifier for mass spectra in ProteomeXchange repositories (including PRIDE).

## TRAINING

Recent years have provided many challenges in terms of training development and delivery, but 2022 saw the reintroduction of in-person training at EMBL-EBI as well as the retention of an extensive virtual programme.

EMBL-EBI’s training programme focuses on empowering scientists to get the most out of openly accessible data resources and services, and to develop key bioinformatics analysis skills. This goal has been supported even further in 2022 through the addition of training in key principles for data management and open data in all EMBL-EBI live courses, as emphasised by EMBL’s updated open science policy.

This enables us to encourage all scientists we train to deposit their data in open resources, ensuring they know how the deposition process works, how to get started and where to find the support required at all points. We are also working with countries where deposition rates have traditionally been low, e.g. LMICs, to determine barriers to deposition and how these can be overcome. Roughly 500 scientists participate in in-person courses per year, with the majority of these reporting that they go on to pass on their learning to others. Web-based on-demand training sees around 500 000 unique IP users per year, of which 80% rate webinars as excellent or very good.

2022 has seen the culmination of a three-year project to completely redesign the training website. Testing indicated a great improvement in user experience for those seeking training courses and content for their own use, or in the training of others. A key development in 2022 was ensuring those undertaking self-directed learning through EMBL-EBI on-demand materials can easily track their progress, keep a record of courses they have completed, and plan their future studies. New Personal Account functionality enables EMBL-EBI trainees to tag favourite courses and manually log their progress through a course, whilst also recording their quiz results and maintaining a record of completion.

New on-demand formats, such as curated collections and learning pathways provide trainees with a more structured approach to learning for a particular topic. Created from a mixture of on-demand tutorials and webinars, alongside excerpts of video and practical exercises from live courses, this guided learning is further reinforced through the EMBL-EBI webinar programme, with the addition of expert panel Q&A sessions.

A final piece of work has been to further improve the FAIRness of EMBL-EBI live training materials, by creating an openly accessible training material set for each course. Materials have always been available via FTP post course, but their reuse was limited as the context of the sessions was often lost. EMBL-EBI new course material sets provide a complete overview of each course and allow for easier reuse by both trainers and trainees.

Finally, EMBL-EBI have set up a trainer-specific space to further build capacity for bioinformatics trainers and educators and support the teaching of EMBL-EBI resources by external trainers using expert written materials.

## CONCLUSION

As the world and the scientific community recover from the ongoing COVID-19 pandemic, there is ample opportunity to reflect on the importance of open science and open data. Open data resources such as those at the EMBL-EBI need to continuously evolve and engage with their user communities to meet changing scientific needs. Many of the developments described above reflect the emerging need to prepare data resources for use in AI applications, which are already starting to transform many scientific fields. Building for AI is reflected in the collection and curation of reference datasets, and the development of community-driven data standards and guidelines that support the reuse of data beyond the bounds of the experiment that generated them.

The transformative potential of AI applications has been amply demonstrated with DeepMind's development of AlphaFold, which predicted protein structure for almost all 200M protein sequences in UniProt, and lead to myriad scientific uses of this new data across many different fields. The data resources at EMBL-EBI can catalyse such developments because they offer sustainable, open availability of high-quality data resources, collected in some cases over decades. Our aim is for EMBL-EBI data resources to keep providing the foundations for tools and research insights that transform fields across the life sciences.

## DATA AVAILABILITY

All of the data resources described above are freely available to access and reuse at https://www.ebi.ac.uk/services.
